# Bridging the Gap between Vision and Progression

**DOI:** 10.3390/cancers16010146

**Published:** 2023-12-28

**Authors:** Boban M. Erovic

**Affiliations:** Institute of Head and Neck Diseases, Evangelical Hospital, 1180 Vienna, Austria; boban.erovic@meduniwien.ac.at

Writing this editorial is more than a great pleasure and honor for me because since its first appearance in 2009, Cancers has become one of the most acknowledged journals among scientists and cancer surgeons. And why? I think one key factor for the success of this journal is that for science and progression, one has to be open-minded for new ideas that subsequently are the basis for innovation and progress.

To be the editor of this special issue was a particular challenge, because as a head and neck surgeon, you have to put a lot of time and effort to keep up with all new scientific tools and pathways to understand the outcome and merit of all presented studies. But in the end, it was more than worth it! Seven studies were published and all papers can be summarized as innovative and visionary.

Han and colleagues could show in this present work that patients with esophageal carcinoma need adjuvant radiotherapy to decrease locoregional failure. Interestingly, the authors could determine that postoperative radiotherapy is effective, however, local-regional recurrence-free survival (LRFS) declined until 50 Gy of dosage was achieved. When 50 Gy were reached the LRA remained steady. In contrast, the hazard-ratio of treatment-related mortality (TRM) was stable until a radiation total dosage (RTD) exceeded 50 Gy, but when this RTD level was passed TRM increased. Furthermore, they could show that a 50 Gy dosage in esophageal patients is well tolerated and most importantly results in a favorable survival with LRFS improvement.

In another review, Wang and coworkers summarized all data regarding immunotherapy as a therapeutical option in benign chordomas. This review confirmed that the use of checkpoint inhibitors like Pembrolizumab, a PD-1/PD-L1 inhibitor, are highly effective with no increase in toxicity in advanced chordoma, and might even be a highly effective option even in PD-L1-negative patients. 

Another immunotherapy study in cetuximab-resistant colorectal cancer (CRC) showed that ACY1, a suggested oncogene, was significantly downregulated in colorectal carcinoma cells after irradiation. Further data analyses showed that ACY1 overexpression was associated with lymph node metastasis, poor prognosis and most interestingly to cetuximab resistance. Subsequently, ACY1 silencing led to decrease in cell proliferation, migration, and invasion abilities but to an enhanced radiosensitivity of these cetuximab-resistant colon carcinoma cell lines after radiotherapy in vitro. This study showed that targeting ACY1 may help in treating cetuximab-resistant colon carcinoma patients. 

To prolong the story with immune-, and targeted cell therapy, Lu and colleagues showed that Taraxasterol (Trax) induced non-small lung cancer cell apoptosis by increasing pro-apoptotic molecules, including Bax, caspase-9, and PARP1 with simultaneous downregulating anti-apoptotic protein Bcl-2 in vitro. Simultaneously, an in vivo tumor model confirmed the in vitro data and showed that Trax upregulates CD107a+ NK cells modulating the tumor microenvironment. The authors concluded that Trax could serve as a new potential natural drug for lung cancer therapy. Another study targeting tumor microenvironment was presented by Shareef and colleagues, who performed a review on the Dickkopf family proteins with a particular focus on Dkk-3. Although Dkk-3-based gene therapy is evolving scarce data on its endogenous role in cancer are available. Dkk-3 has been found in the tumor environment, in particular in fibroblasts and endothelial, and immune cells, in close neighborhood to epithelial cells. Buczynska and colleagues showed that metformin, commonly used for diabetes mellitus therapy, provides anti-inflammatory, anti-cancer, hepato-, cardio-, oto-, radioprotective and radio-sensitizing properties, that should be translated into human trials in the near future. Money and colleagues made the bridge from treating patients with immunotherapy and plant-based drugs to alternative therapies. This review showed that under-recognized adjunctive therapies like massage, yoga, Tai-Chi, breathing exercises, good sleep quality and diet modifications can help to improve cancer therapy significantly. Moreover, supplements like aspirin, green tea, turmeric, and melatonin should be tailored to the individual patient demands, first to assure/ensure no side effects and secondly to prevent negative influence on administered immune or chemotherapy.

Summarized, all presented studies are excellent examples of bridging evidence-based science to clinical practice. All presented topics are trying to merge and focus newly gained knowledge to improve outcome of cancer patients and are prime examples to motivate all of us with the ultimate aim to conquer cancer.

